# Impact of icing weather conditions on the patients in helicopter emergency medical service: a prospective study from Northern Finland

**DOI:** 10.1186/s13049-019-0592-8

**Published:** 2019-02-12

**Authors:** Ilkka Pulkkinen, Jari Pirnes, Ari Rissanen, Päivi Laukkanen-Nevala

**Affiliations:** 1 FinnHEMS Research and Development Unit, Vantaa, Finland; 2 FinnHEMS 51, Lapland HEMS Unit, Lentoasemankuja 18, 96930 Rovaniemi, Finland; 3 Department of Anaesthesia and Intensive Care, Länsi-Pohja Central Hospital, Kemi, Finland

**Keywords:** Delay in definitive treatment, Helicopter emergency medical service (HEMS), Icing weather conditions

## Abstract

**Background:**

A high number of denied or cancelled HEMS missions are caused by poor weather conditions especially during winter season. Furthermore, many helicopter manufacturers have denied their helicopters to be operated in known icing conditions. Icing is a widely known phenomenon in aviation, but there is a lack of evidence about its influence on HEMS operations and patients.

**Methods:**

A prospective observational study of HEMS missions in Northern Finland was conducted over a 1-year period in 2017. A patient was included in the study when the use of helicopter was denied or cancelled due to icing weather conditions. Patients were categorised into two groups based on whether definitive treatment was delayed or not according to previously defined end-points.

**Results:**

During the study period the Finnish northernmost HEMS unit received 1940 missions. A total of 391 missions (20%) could not be operated by helicopter because of poor weather conditions. In 142 of these missions (36%) icing was one of the limiting weather factors. The year-round incidence of icing was 7.3/100 missions. A total of 57 patients were included in the analysis. Icing weather conditions, resulting in denied helicopter flights, caused a delay in definitive treatment for 21 patients (37%). Definitive treatment was more often delayed in trauma and internal medicine patients than in neurological patients. Nevertheless, the patients whose definitive treatment was delayed were located closer to the hospital. The estimated time that would have been saved by helicopter transport was more than 60 min for 10 patients with delayed treatment.

**Conclusions:**

In this study the incidence of icing weather conditions was substantial compared to all HEMS missions in year 2017. The delay in definitive treatment was accentuated among trauma and internal medicine patients. During the 1-year study period many patients whose definitive treatment was delayed would have had a notable (> 60 min) time saved by helicopter transport. A helicopter equipped with an adequate ice protection system for the weather conditions in Northern Finland would have shortened the delay in patients’ definitive treatment significantly.

## Background

It has been shown that air medical transport improves survival of many patients with medical and trauma emergencies [1], yet overall more than one third of all helicopter emergency medical service (HEMS) missions are denied or cancelled [1–3]. A significant percentage of denied or cancelled HEMS missions is caused by poor weather conditions [1, 3, 4]. Nevertheless, the role of icing weather conditions is unknown within denied or cancelled HEMS missions.

Many helicopter manufacturers have denied their helicopters to be operated in known icing conditions and have instructed pilots to avoid icing environment due to significant safety risks and negative effects on helicopter performance. However, modern helicopters are widely used in HEMS operations that require flying in instrument meteorological conditions (IMC) and in marginal visual meteorological conditions (VMC). These factors increase the danger of inflight icing if entering visible moisture conditions at low temperatures [5, 6].

The Finnish HEMS system is managed by a national administrative company FinnHEMS Ltd. and there are six HEMS units available 24/7. Five HEMS units are physician-staffed and the northernmost unit is manned with advanced-level paramedics. Three southern bases use Airbus H135 and three northern bases use Airbus H145 helicopters. Both helicopter types are certificated for non-icing meteorological conditions only [7, 8]. A rapid response car (RRC) is available in every base for short-range missions and for poor weather conditions. The HEMS units in Finland are dispatched via Finnish Emergency Response Centre (112 Finland).

This study was performed in Northern Finland (Finnish Lapland) where the northernmost HEMS unit FinnHEMS 51 (FH51) operates. The Finnish Lapland accounts for almost 30% of the country’s area. The region is remote and sparsely habited, covering only 3% of the Finnish population [9]. There are 5 airfields in the area that are equipped for instrument flight rules (IFR) operations. FH51 is based at Rovaniemi airport where air-traffic control services are available 24/7. Other airfields have limited availability for those services, which currently reduces the possibilities for IFR missions. The Emergency Medical Services (EMS) in the area is a three-level system with first response units, basic-level and advanced-level ambulances. The paramedics of FH51 are equipped to give more treatments than the personnel of advanced-level ambulances. Physician-staffed HEMS unit (FH50) is located within the Oulu University Hospital and offers consultation services 24/7.

The main hospital in the region is Lapland Central Hospital in the city of Rovaniemi. The longest air distance to the hospital from the northernmost borderline is 411 km. In Central Hospital percutaneous coronary intervention (PCI) is immediately available during office hours. During night time and weekends the availability of PCI is limited. Computed tomography and acute ischaemic stroke thrombolysis are available 24/7 [10]. The nearest university hospital is located in the city of Oulu, 166 km (air distance) south from the Lapland Central Hospital. Oulu University Hospital is a tertiary level teaching hospital that currently provides treatment for people in Northern Finland in general. Round-the-clock exacting special health care is centralized to this university hospital, including PCI treatment, thrombectomy, neurosurgery, cardiothoracic surgery, vascular surgery, hand surgery, paediatric and neonatal intensive care and the treatment of multi-trauma patients [11].

Because of long distances, challenging weather conditions and centralized exacting special health care in the area, the year-round use of helicopter is important for the patients. The aim of this study was to describe the incidence of icing weather conditions and the subsequent delays in patients’ definitive treatment in Northern Finland HEMS missions. Considering how these factors and other weather-related flight restrictions affect the patients, we also assessed the usefulness of a HEMS helicopter with a de-icing system.

## Methods

### Study design

In this observational prospective study, we included all patients from requested FH51 missions where helicopter could not be used due to icing weather conditions between 1st of January and 31st of December 2017. Rapid response car missions were included if the reason to use a RRC was icing weather conditions. Missions were excluded from the study when a patient died at the scene, was treated at the scene by the local Ground Emergency Medical Service (GEMS) unit without hospital transport or was transported to the regional health care facility. Secondary transfers of the already included patients were excluded. In addition, missions in which weather was below minima or air-traffic control services were not available at the time of dispatch were excluded.

Delay in definitive treatment was defined by the following four end-points: 1) patient died within 24 h, 2) patient was transported to university hospital within 24 h, 3) the time frame for stroke treatments (thrombolysis 4.5 h or thrombectomy 6 h) was missed, or 4) patient was transferred immediately after emergency department (ED) admission to the ICU, PCI treatment or operating theatre.

The end-points were defined based on evidence of morbidity and mortality about critical patients and according to Finnish Current Care Guidelines. It has been shown that survival from severe traumatic haemorrhage and traumatic brain injury is poor and considerable proportion of those patients dies within 24 h [12]. The time frame for stroke treatments is defined by Finnish Current Care Guidelines [13]. Increased prehospital delays before arriving to percutaneous coronary intervention-capable hospital is associated with higher mortality and morbidity [14]. Among internal medicine patient’s, the number of sepsis deaths is rising due to increasing incidence [15]. The EMS personnel plays key roles in improving survival of sepsis patients with early prehospital recognition, initial resuscitative therapy and rapid transport to nearest appropriate receiving hospital [15, 16].

The study was approved by the administration of Lapland Hospital District (TUT 40/2016, 27th Oct 2016) and the local ethics committee (Eettmk §337/14th Nov 2016).

### Study setting

We used a structured questionnaire for data collection. When icing was the reason to deny or cancel a helicopter mission, the HEMS paramedic on duty made a phone call to the local GEMS unit at the scene and registered the patient’s social security number and destination for the GEMS transport. On missions responded with RRC by the FH51 medical crew, patient’s social security number was collected from the EMS documents. Patient data were retrieved later from the hospital electronic patient record system, EMS database (Codea database version 1.33) and EMS documents using social security numbers. Icing weather conditions were registered in FinnHEMS database that was developed further for the study purposes.

After a helicopter flight was denied due to icing weather, the pilots on duty re-reviewed the actual and forecasted aviation weather covering the region needed for the mission. The meteorological data of the Finnish Meteorological Institute was used for the weather review. Based on the available weather information, the pilots assessed the feasibility of performing the mission by helicopter if all the icing conditions in the weather data were ignored. Other weather limitations than icing, as well as the availability of air-traffic control services at the region, were considered as they were issued. Pilots also estimated different possibilities to execute the flight according to the flight planning weather minima described in the Operations Manual of the airline company (Babcock Scandinavian Air Ambulance), with the icing conditions excluded from the weather data. Flights were classified into three categories based on estimated flight procedures: 1) totally visual flight rules (VFR) flight, 2) partly VFR and partly IFR flight (e.g. IFR departure from the base and VFR landing at the scene) or 3) totally IFR flight (airport to airport).

Demographic data included the patient’s age, gender and the American Society of Anesthesiologists (ASA) physical status classification. Based on the reason for the HEMS mission, the patients were classified into three categories: internal medicine, neurology and trauma. The ICD-10 codes of the treatment period at issue were recorded. Mission date, dispatch time, address, beginning of GEMS transport time, GEMS arrival time at the hospital, actual transport time (from the beginning of patient transport on scene to arrival at destination hospital), priority of transport and the transport code were collected. Use of RRC or other aircraft was recorded. The distance from scene to destination hospital was registered by road and by air using a map software (Reittikartta Suomi version 2.3.0). The estimated transport time by helicopter from the scene to the destination hospital was calculated by using airspeed 135 knots (250 km/h).

### Statistical analysis

We designed this study to be descriptive and observational. Based on the number of denied and cancelled missions due to poor weather conditions (mean, 357/year) during 2012–2016, we considered 1-year data collection to provide a sufficient number of missions. The data were checked twice by two persons to avoid human error. Results are expressed as counts, incidences (with 95% confidence intervals [CIs]), means and standard deviations or percentages, depending on the variables. The chi-square test and Fisher exact test were used for categorical data. The continuous data was analysed using the independent samples *t* test. Data were analysed with SPSS (IBM SPSS statistics version 24). A *p* value of < 0.05 was considered statistically significant.

## Results

**Fig. 1:**
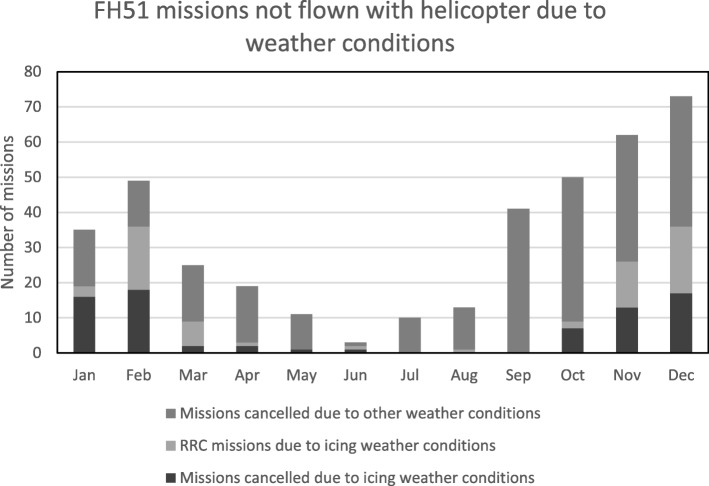
FinnHEMS 51 missions not flown with helicopter due to weather conditions in 2017: missions cancelled due to icing weather conditions or other weather conditions and RRC missions due to icing weather conditions

During the study period FH51 received 1940 missions. A total of 391 missions (20%) could not be operated by helicopter because of poor weather conditions. Of these, in 142 (36%) missions icing was one of the limiting weather factors. The year-round incidence of icing was 7.3/100 missions (95% CI 6.2–8.5), which was 1.46 times (95% CI 1.25–1.72) more than the incidence of icing in all three northern HEMS bases (5.0/100 missions, 95% CI 4.5–5.5). Icing weather conditions were accentuated in the winter months (January, February and December); there were in total 157 missions not flown due to poor weather conditions during this three-month period, and 91 (58%) of these were helicopter flights denied due to icing weather (Fig. 1).

**Fig. 2:**
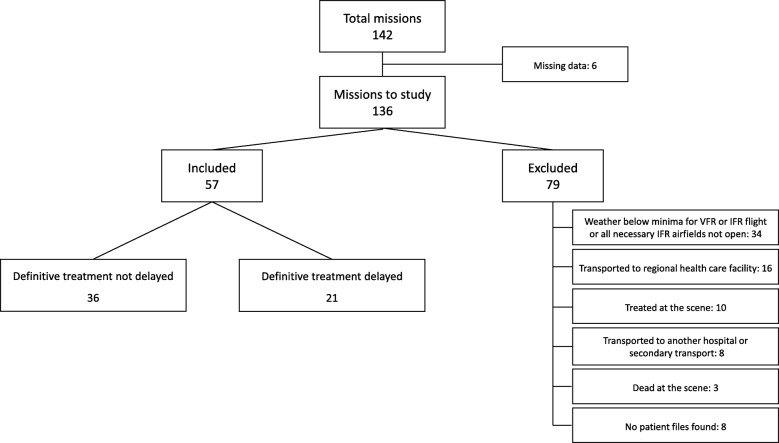
Flowchart

We received 136 filled study forms during the study period. Six study forms (4.2%) were missing. A total of 57 patients were included in the analysis and 79 patients were excluded based on the exclusion criteria. According to the predefined end-points, definitive treatment was delayed in 21 patients (37%). Of those, 8 patients were transferred immediately after central hospital ED admission to ICU and 3 patients for PCI treatment. Two of the ICU patients were operated after stabilization of vital functions. Eight patients were transported to the university hospital. Of those, 3 patients were transported straight from the scene, 2 patients by another helicopter and 1 patient by GEMS. Two patients died within 24 h in the central hospital (Fig. 2, Table 1).

**Table 1:** Report of 21 patients with estimated delay in definitive treatment

Patient	Age (years)	Gender	ASA	Type of mission	Type of transport	Road distance to hospital (km)	Actual transport time (min)	Estimated saving of time by helicopter transport (min)	Estimated (or actual) flight procedure	Diagnosis
Immediate move to ICU, PCI or surgical operation										
#1	45	Female	1	Snowmobile accident	BG helicopter	179	50	Transported by helicopter	Actual VFR and IFR	Subarachnoid haemorrhage and pulmonary contusion
#2	80	Male	3	Unconsciousness	Ambulance and RRC	153	80	48	VFR	Unconsciousness of occlusion and acidosis
#3	64	Male	2	Chest pain	Ambulance and RRC	135	63	42	VFR	STEMI, primary PCI
#4	40	Female	2	Downhill skiing accident	Ambulance and RRC	174	121	85	VFR and IFR	Kidney injury
#5	75	Male	3	Dyspnoea	Ambulance and RCC	167	115	84	VFR and IFR	Pneumonia with RS-virus
#6	77	Male	3	Chest pain	Ambulance and own HEMS unit	330	229	Transported partly by helicopter	Actual VFR	STEMI, rescue PCI
#7	68	Male	3	Unconsciousness	Ambulance and RRC	154	121	89	IFR	Basilar artery thrombosis and brainstem stroke
#8	73	Female	2	Chest pain	Ambulance and RRC	118	72	49	VFR	STEMI, primary PCI
#9	78	Female	2	Cardiac arrest	Ambulance and RRC	105	71	50	VFR and IFR	Cardiac arrest, ROSC in 20 min
#10	81	Male	3	Stroke	Ambulance	260	151	97	VFR and IFR	Intracerebral haemorrhage
#11	78	Male	2	Chest pain	Ambulance and RRC	172	108	72	IFR	Recurrent ventricular tachycardia
Transfer to university hospital within 24 h										
#12	18	Male	1	Snowmobile accident	BG helicopter	360	77	Transported by helicopter to UH	Actual VFR and IFR	Paraparesis
#13	61	Male	3	Emergency transfer to UH	Ambulance	226	117	77	VFR and IFR	Cardiac tamponade, immediate thoracotomy at UH
#14	29	Male	1	Traffic accident	Ambulance and another HEMS unit	473	200	Transported partly by helicopter to UH	Actual VFR	Pelvic and thigh contusion
#15	74	Female	2	Chest pain and unconsciousness	Ambulance and RRC	102	77	56	VFR and IFR	Aortic dissection, type A
#16	64	Male	2	Tractor accident	Ambulance and RRC	28	24	20	VFR and IFR	Critical hand injury
#17	17	Female	1	Snowmobile accident	Ambulance and RRC	227	172	128	VFR and IFR	Multi-trauma patient
#18	75	Female	2	Traffic accident	Ambulance and RRC	146	93	69	VFR and IFR	Multi-trauma patient
#19	15	Male	1	Snowmobile accident	Ambulance	228	120 (to UH)	78	VFR and IFR	Amputation of hand
Dead within 24 h										
#20	55	Female	3	Cardiac arrest	Ambulance and RRC	133	75	49	VFR and IFR	Cardiac arrest, ROSC in 17 min
#21	81	Female	3	Unconsciousness	Ambulance and RRC	134	134	108	VFR and IFR	Subarachnoid haemorrhage

*ASA* American Society of Anesthesiologists physical status class, *BG* Border Guard, *ICU* intensive care unit, *IFR* instrument flight rules, *PCI* percutaneous coronary intervention, *ROSC* return of spontaneous circulation, *RRC* rapid response car, *STEMI* ST-elevation myocardial infarction, *UH* university hospital, *VFR* visual flight rules

Sixty-seven percent of missions were dispatched in the daytime (from 8 a.m. to 8 p.m.). Forty-eight percent of the missions in which the patient’s definitive treatment was delayed were dispatched during the night time (from 8 p.m. to 8 a.m.), but the difference was not statistically significant (*p* = 0.14). In 65 missions the RRC was used as a substitute for helicopter because of icing weather, but 38 of those missions (58%) were cancelled during the drive to the scene. In the remaining 27 missions the patient was encountered by the FH51 medical crew with RRC and accompanied by the HEMS paramedic with local GEMS unit to the hospital. In 14 of those missions (52%) the patient’s definitive treatment was delayed. Seven (12%) of the included 57 missions were operated partly by using a helicopter. During the study period four patients were transported by a Finnish Border Guard helicopter, one patient by a Finnish Defence Forces helicopter and one patient by another HEMS helicopter. One mission was operated with the HEMS unit’s own helicopter after the sunrise when there was enough daylight for a VFR flight. The use of RRC and the use of another helicopter were more frequent with patients with delay in definitive treatment (*p* = 0.005). Twenty-eight patients (49%) were transported with highest priority and 18 (64%) of those patients were estimated to suffer a delay in definitive treatment according to the (pre-defined) end-points (*p* < 0.001) (Table 2).

**Table 2:** Data on dispatch time and patient transport

Characteristic	All patients (*n* = 57)	Definitive treatment not delayed (*n* = 36)	Definitive treatment delayed (*n* = 21)	*p* value
Dispatch time, n (%)				0.14^a^
8 a.m. – 8 p.m.	38 (67)	27 (75)	11 (52)	
8 p.m. – 8 a.m.	19 (33)	9 (25)	10 (48)	
Type of transport, n (%)				0.005^b^
Ambulance	23 (40)	20 (56)	3 (14)	
Ambulance and RRC	27 (47)	13 (36)	14 (67)	
Ambulance and helicopter	7 (12)	3 (8)	4 (19)	
Code of transport, n (%)				0.10^b^
Stroke	14 (25)	13 (36)	1 (5)	
Motor vehicle accident				
Traffic	4 (7)	2 (6)	2 (10)	
Off-road	6 (11)	3 (8)	3 (14)	
Mechanical injury	5 (9)	2 (6)	3 (14)	
Chest pain	7 (12)	4 (11)	3 (14)	
Unconsciousness	4 (7)	1 (3)	3 (14)	
Cardiac arrest	3 (5)	1 (3)	2 (10)	
Other medical symptoms	9 (16)	6 (17)	3 (14)	
Transfer to tertiary care	5 (9)	4 (11)	1 (5)	
Priority of transport, n (%)				< 0.001^b^
A (highest)	28 (49)	10 (28)	18 (86)	
B	25 (44)	22 (61)	3 (14)	
C	4 (7)	4 (11)	0	
D (lowest)	0			

*RRC* rapid response car

^a^ Chi-square test, ^b^ Fisher exact test

The patients’ mean age was 57.0 years and 51% of the patients were in the age group of 18–65 years. The biggest patient group consisted of neurological patients (39%), but a delay in definitive treatment was most frequent in trauma and internal medicine patients (86% of all patients with delayed definitive treatment, *p* = 0.016) (Table 3).

**Table 3:** Demographic data, ASA classification and patient classification categorised according to delay in definitive treatment

Characteristic	All patients (*n* = 57)	Definitive treatment not delayed (*n* = 36)	Definitive treatment delayed (*n* = 21)	*p* value
Age, mean (SD)	57.0 (21.9)	55.5 (21.5)	59.4 (22.8)	0.52^a^
Age group, n (%)				0.36^b^
< 18 yrs	4 (7)	2 (6)	2 (10)	
18–65 yrs	29 (51)	21 (58)	8 (38)	
> 65 yrs	24 (42)	13 (36)	11 (52)	
Gender (male), n (%)	30 (53)	18 (50)	12 (57)	0.78^c^
ASA classification, n (%)				0.89^b^
ASA I	15 (26)	10 (28)	5 (24)	
ASA II	18 (32)	10 (28)	8 (38)	
ASA III	23 (40)	15 (42)	8 (38)	
ASA IV	1 (2)	1 (3)	0	
Patient classification, n (%)				0.016^c^
Internal medicine	20 (35)	10 (28)	10 (48)	
Neurology	22 (39)	19 (53)	3 (14)	
Trauma	15 (26)	7 (19)	8 (38)	

^a^ Independent samples t test, ^b^ Fisher exact test, ^c^ Chi-square test

### Subgroup analysis of ground unit missions

Missions where no helicopter was able to fly were analysed in their own subgroup. This subgroup included 50 missions (88%) with GEMS or GEMS and RRC together.

In this subgroup the patients whose definitive treatment was delayed were located closer to hospital compared with patients whose definitive treatment was not delayed, 157 vs. 200 km by road (*p* = 0.028) and they achieved hospital earlier, 101 vs. 128 min (*p* = 0.032), respectively. The average transport time by GEMS to hospital was almost 2 h (119 min). The estimated average transport time by HEMS was 36 min in this subgroup. The estimated average HEMS transport time would have been 82 min shorter than the actual average GEMS transport time was. For 74% of the patients the estimated time saved would have been more than 60 min if they had been transported by the HEMS unit. Of those, 10 patients’ (59%) definitive treatment was estimated to be delayed. There was no significant difference between the groups (*p* = 0.17) (Table 4).

**Table 4:** Distance, transport time and estimated flight procedure. Those missions in which the patient was transported partly by helicopter (*n* = 7) were excluded from the table

Characteristic	All patients (*n* = 50)	Definitive treatment not delayed (*n* = 33)	Definitive treatment delayed (*n* = 17)	*p* value
Distance to hospital (km), mean (SD)				
By road	186 (68)	200 (69)	157 (57)	0.028^a^
By air	152 (55)	165 (55)	126 (48)	0.017^a^
Transport time (min),mean (SD)				
GEMS (actual)	118 (43)	128 (43)	101 (36)	0.032^a^
HEMS (estimated)	36 (13)	40 (13)	30 (12)	0.017^a^
Difference GEMS vs. HEMS	82 (33)	88 (34)	71 (27)	0.067^a^
Estimated saving of time by HEMS, n (%)				0.17^b^
< 60 min	13 (26)	6 (18)	7 (41)	
60–90 min	22 (44)	15 (46)	7 (41)	
> 90 min	15 (30)	12 (36)	3 (18)	
Estimated flight procedure, n (%)				0.63^b^
VFR completely	9 (18)	5 (15)	4 (23)	
Partly VFR and partly IFR	32 (64)	21 (64)	11 (65)	
IFR completely	9 (18)	7 (21)	2 (12)	

*GEMS* ground emergency medical service, *HEMS* helicopter emergency medical service, *IFR* instrument flight rules, *VFR* visual flight rules

^a^ Independent samples t test, ^b^ Fisher exact test

Most (82%) of the included 50 missions in this subgroup could have been operated either completely by VFR flights or partly by VFR and partly by IFR flights. Also, the majority of missions (88%) in which the patient’s definitive treatment was delayed could have been performed with VFR flights or with partly VFR/partly IFR flights. Only 9 missions (18%) could have been completed only by IFR flights. In 8 of those, the closest IFR airport would have been the same one, located at a mean distance of 34 km by road from the scenes (Table 4).

## Discussion

There are four main results to be presented in our study. First, the incidence of icing weather conditions in HEMS operations in Northern Finland was high. Second, icing weather caused a delay in definitive treatment more often for trauma and internal medicine patients than for neurological patients. Third, even though the patients whose definitive treatment was delayed (59%) were located closer to hospital than the patients whose definitive treatment was not delayed, the estimated time that would have been saved with helicopter transport was more than 60 min. Finally, the FH51 medical crew’s advanced care with RRC was necessary to patients whose definitive treatment was delayed, but the delay in definitive treatment was notable when using RRC compared to helicopter.

Poor weather conditions have been reported to frequently cause denied or cancelled HEMS and search and rescue (SAR) missions [1–4, 17]. Studies have reported that 5.1–9.7% of all requested HEMS missions are denied or cancelled yearly due to poor weather conditions with the rotor wing aircraft [1, 2]. In our study, on average 20% of missions were not flown due to poor weather conditions, and during winter time the percentage of missions not flown was up to 40%. Previous studies in areas with similar seasons have reported parallel findings [1, 2, 4]. A Norwegian study of three HEMS bases reported that the proportion of missions that were denied or cancelled due to poor weather conditions increased from 5.1 to 8.4% during the winter months, making it 1.6 times greater than the annual average of missions denied or cancelled due to weather conditions [2]. A previous study from South East England also showed that the number of night-time HEMS missions that were denied because of poor weather conditions increased during the winter months [4].

Although icing is a widely known phenomenon in aviation, there is a lack of evidence about its influence on HEMS operations. The incidence of icing weather conditions was notably higher in the northernmost HEMS base compared with two other northern HEMS bases in Finland. According to our results, most of the missions could have been operated completely by VFR or by a combination of VFR/IFR flights by a helicopter equipped with an adequate ice protection system for the weather conditions in Northern Finland. These results are reinforced by the few helicopter missions that were operated by the Finnish Border Guard helicopter and the Finnish Defence Forces helicopter. The Defence Forces helicopters (NH90) have an ice protection system on board, and both organizations have lower weather minima than commercial helicopter providers such as HEMS operators.

In our study, the delay in definitive treatment was most frequent among trauma and internal medicine patients. One explanation for this finding could be the centralization of multi-trauma and traumatic brain injury treatments to the university hospital. Internationally, seriously injured patients seem to have a higher mortality in rural and sparsely habited areas than in urban areas [18, 19]. In Western Australia the death rate among patients with major trauma in rural areas has been reported to be over four times the rate in major cities. However, in those patients in rural areas who survive to be retrieved to a tertiary hospital by air transport the mortality outcomes are equivalent to the metropolitan area in Western Australia [19]. Several studies suggest that air transport improves the chance of survival in certain trauma patients when the patient is treated by the HEMS crew and transported to the final destination hospital by helicopter [20–23].

In our study population, three patients with ST-elevated myocardial infarction (STEMI) were transferred directly to a medical facility for PCI treatment. One of these patients was transferred for rescue PCI treatment because of ineffective thrombolysis treatment. Lapland Central Hospital was able to activate a PCI team for every cardiac patient in our study, and no patients were transferred to university hospital for PCI treatment. In Canberra region in Australia a pre-hospital diagnosis of STEMI and direct transfer to PCI capable hospital for coronary angiography reduced total ischaemic time by almost an hour and significantly reduced mortality following primary PCI treatment [14]. In rural areas the use of helicopter transport may improve cardiac patients’ access to primary PCI treatment.

During the study period no acute stroke treatment was missed even though the biggest patient group in the study population consisted of neurological patients. A previous study in two hospital districts in Northern Finland showed that in the light of results from a risk assessment with National Early Warning Score (NEWS) there is a significant overtriage in two of the highest dispatch priorities assessed by the Finnish Emergency Response Centre [24]. The overtriage may partly explain our finding that definitive treatment was less often delayed among neurological patients than in the other patient groups. Nevertheless, helicopter transport is important for some patients with acute neurological disorders in Northern Finland because of the long distances in this area; reaching available necessary care, such as acute ischaemic stroke thrombolysis treatment, within a critical time window may be possible only with air medical transport. Indeed, in the United States the HEMS providers have been proven to increase patients’ timely access to definitive stroke care in rural and super-rural areas [25].

In our results, the majority of patients whose definitive treatment was delayed were located closer to the receiving hospital than the patients whose definitive treatment was not delayed. One explanation for this could be that the density of population is higher within a radius of approximately 100 km from Rovaniemi city area. Another explanation could be that there are a few popular tourism villages and ski resorts located approximately 170 km north of the FH51 base. Nevertheless, the estimated time saved by helicopter transport was significant for the patients whose definitive treatment was delayed. In Oklahoma, United States, more than 80% of injured patients were transported by a helicopter to the receiving level I or level II trauma centre when the distance from the scene was more than 56,3 km. After the decision to transport a trauma patient directly from the scene to a trauma care centre, the distance was the main factor in deciding whether to use a ground unit or a helicopter [21]. In those of our study patients whose definitive treatment was delayed the mean air distance from the scene to the receiving hospital was more than two times longer (126 km) than that in the study from Oklahoma. In rural areas the ability to fly a helicopter in different weather conditions may have a higher role in patient survival than in urban areas. Furthermore, sparse population, long distances and prolonged ground transport to the hospital are common reasons to use air transport for patients [26].

The use of RRC was well adapted to its purpose as a substitute for helicopter in case of poor weather conditions especially in the winter months. The patients accompanied by a HEMS paramedic to the hospital were more often critically ill or injured than those patients who were transported by GEMS alone, and the use of RRC advanced the beginning of definitive prehospital care. In a Norwegian study on the use of RRC as a supplement to ambulance helicopter, 224 of the 605 diagnosed patients (37%) received advanced-level medical treatment that was not generally available in the prehospital setting of that area [3]. This finding is in accordance with our results. In our study, 58% of RRC missions as a substitute for helicopter were cancelled. Long distances in a sparsely habited area and the possible overtriage in HEMS dispatches may explain the high rate of cancellations. Despite the advanced level of care by FH51 medical crew with RRC in the not-flown missions, the benefits of time saved by helicopter transport still would have been substantial and in some cases crucial.

There are some limitations to this study. First, we cannot guarantee that all denied or cancelled missions due to icing weather conditions were registered in the FinnHEMS Database; thus, more than six study forms could be missing. Second, the study was carried out at only one HEMS base with a limited and sparsely habited area. Third, the sample size was limited because of the predefined 1-year data collection. Fourth, since this was an observational study the results can only indicate possible associations, not causal relationships.

## Conclusion

In this study the incidence of icing weather conditions was 7.3/100 HEMS missions. Icing weather conditions caused delay of definitive treatment more often for trauma and internal medicine patients than for neurological patients. For 10 patients whose definitive treatment was delayed during this 1-year study period the time saving with air medical transport would have been more than 60 min if icing conditions had not limited the use of a helicopter. A helicopter equipped with an adequate ice protection system for weather conditions in Northern Finland would have decreased the delay in patients’ definitive treatment significantly.
